# Editorial—Current Insights on Lipid-Based Nanosystems

**DOI:** 10.3390/ph15101267

**Published:** 2022-10-14

**Authors:** Ana Catarina Silva, João Nuno Moreira, José Manuel Sousa Lobo

**Affiliations:** 1 UCIBIO/REQUIMTE, MEDTECH, Laboratory of Pharmaceutical Technology, Department of Drug Sciences, Faculty of Pharmacy, University of Porto, 4050-313 Porto, Portugal; 2 FP-I3ID (Instituto de Investigação, Inovação e Desenvolvimento), FP-BHS (Biomedical and Health Sciences Research Unit), Faculty of Health Sciences, University Fernando Pessoa, 4249-004 Porto, Portugal; 3 CNC—Center for Neurosciences and Cell Biology, Center for Innovative Biomedicine and Biotechnology (CIBB), Faculty of Medicine (Polo 1), University of Coimbra, Rua Larga, 3004-504 Coimbra, Portugal; 4 Pólo das Ciências da Saúde, Faculty of Pharmacy, CIBB, Univ. Coimbra—University of Coimbra, Azinhaga de Santa Comba, 3000-548 Coimbra, Portugal

Lipid-based nanosystems, including solid lipid nanoparticles (SLN) and nanostructured lipid carriers (NLC), cationic lipid nanoparticles, nanoemulsions and liposomes, have been extensively studied to improve drug delivery through different administration routes. The main advantages of these systems are the ability to protect, transport and control the release of lipophilic and hydrophilic molecules (either small-molecular-weight molecules and macromolecules), the use of generally recognized as safe (GRAS) excipients that minimize the toxicity of the formulations, and the possibility to modulate pharmacokinetics and enable the site-specific delivery of encapsulated payloads. In addition, the versatility of lipid-based nanosystems has further been demonstrated for the delivery of vaccines, protection of cosmetic actives, and improvement of moisturizing properties of cosmetic formulations.

Currently, lipid-based nanosystems are well established and there are already different commercially approved formulations in different human disorders. This success has actually paved the way to diversify the pipeline of development, to address unmet medical needs for several indications, such as cancer, neurological disorders, and autoimmune, genetic and infectious diseases.

This Special Issue aims to update readers on the latest research on lipid-based nanosystems, both at the preclinical and clinical levels. A series of 15 articles (six reviews and nine studies) is presented, with authors from 12 different countries, showing the globality of the investigations that are being carried out in this area.

Ana Catarina Silva et al. [1] revised the state of the art of in vitro cell models to perform studies with drug-loaded SLN and drug-loaded NLC nasal formulations. The authors concluded that specific in vitro cell culture models, such as the human nasal epithelial cells (HNEpC) and the human nasal septum quasidiploid tumour cells (RPMI 2650), are needed to assess the cytotoxicity of nasal formulations and to understand the mechanisms of nasal drug transport and absorption. In addition, the authors reported the great potential of using 3D nasal casts to test the effectiveness of formulations for reaching the upper part of the nasal cavity, which is critical for successful nose-to-brain delivery. These models are manufactured from computed tomography scans of the human nasal cavity and enable the analysis of the factors interfering with nasal drug deposition, including the nasal cavity area, type of administration device and angle of application, inspiratory flow rate, among others. Although 3D models are already being used to test nasal formulations, this remains an open field of research, as their validation by regulatory authorities is required.

Habibah A. Wahab et al. [2] highlighted the potential of lipid-based nanocarriers for pulmonary drug delivery for the treatment of lung cancer. The authors presented considerations of the physiological, physicochemical and technological aspects of efficient inhalable anticancer drugs delivery systems based on lipid-based nanocarriers, and their pioneering role in the treatment of lung cancer. Lipid-based nanocarriers are able to transport drugs with different physicochemical characteristics, show enhanced permeability and retention effects for passive targeting, and can be functionalized to provide active targeting. Recent preclinical studies show that inhalable lipid-based nanocarriers can be concentrated in the lungs, from where they diffuse into the blood stream and lymphatic system, reaching cancer cells. However, the authors point out that research in this area needs to advance towards in vivo and clinical studies. In the field of anticancer therapies, Vijay Gyanani et al. [3] presented a brief review on the challenges of conventional therapies and the use of liposomes as a targeting strategy for the delivery of anticancer drugs. The authors discussed the challenges and limitations of conventional anticancer treatments (chemotherapy, radiotherapy and surgery), such as drug resistance, severity and side effects, and highlighted future research opportunities in this field. The use of active targeted liposomes remains challenging due to antigen and receptor heterogeneity, immunogenicity and low drug encapsulation efficiency, while passive targeted liposomes overcome the limitations of immunogenicity. However, there are manufacturing and clinical challenges associated with active and passive liposomes, primarily related to translating preclinical into clinical efficacy.

Viliana Gugleva et al. [4] provided a review of the therapeutic properties of different classes of phenolic compounds used for dermal application, including their effects on oxidation processes, inflammation, vascular pathology, immune response, precancerous and oncological lesions or formations, and microbial growth. The authors also presented several examples of promising results from studies with phenolic compounds encapsulated in lipid-based nanocarriers (nanoemulsions, liposomes, SLN and NLC), which aimed to improve their solubility, stability, skin permeation and therapeutic activity. On the use of lipid-based nanocarriers to improve drug delivery to the skin, Stefanov and Velichka Y. Andonova [5] presented the state of the art on recent developments in the application of these nanocarriers in topical formulations to treat skin disorders as an alternative to the conventional formulations, reducing systemic toxicity. So far, lipid-based nanocarriers have been shown to provide a flexible platform for safe, effective and biocompatible topical drug delivery, as they do not cause cytotoxicity or morphological changes through the skin.

Stephan T. Stern et al. [6] described current trends in the development of the next generation of tissue-targeted lipid nanoparticles containing nucleic acids for different therapeutic applications, including cancer, and neurological, cardiovascular and infectious diseases. The researchers underlined the interest of using lipid nanoparticles to actively target nucleic acids to the vascular endothelium through receptor-mediated transcytosis and paracellular transport, which must rely on tissue-selective receptor expression that can be identified through modern ligand–receptor identification techniques, such as phage display. Tissue targeting can be achieved by manipulating the composition of lipid nanoparticles, binding to targeting ligands or introducing cell membrane-derived components into the nanoparticles. In addition, modification in lipid components or nucleic acid cargo can be used to prepare stimuli-responsive lipid nanoparticles that react to internal or external stimuli to release their cargo.

Ehsan Ahmadpour et al. [7] evaluated in vitro the scolicidal and apoptotic activity of liposomes loaded with juglone (5-hydroxy-1,4-naphthoquinone) against protoscoleces, a larval stage of the cestode *Echinococcus granulosus* that causes helminth diseases. All tested concentrations of juglone-loaded liposomes induced scolicidal effects, although only concentrations of 800 μg/mL and 400 μg/mL induced 100% mortality. Furthermore, caspase-3 mRNA expression was higher after exposure with juglone-loaded liposomes compared to the control. From these findings, the authors concluded that optimal doses of juglone-loaded liposomes have potent scolicidal effects on the *Echinococcus granulosus* cestode, although this evidence must be confirmed in vivo.

Ildikó Csóka et al. [8] demonstrated in vitro the advantages of combining SLN and hydrogels to improve the intranasal delivery of antioxidants, increasing absorption and residence time in the nasal mucosa. The researchers used the quality-by-design (QbD) approach and the central composite design to optimize a chemically linked hyaluronic acid hydrogel containing n-propyl gallate-loaded SLN for intranasal delivery as a promising alternative for the treatment of brain tumours, such as glioblastoma multiforme, avoiding the need to cross the blood–brain barrier and improving patients’ compliance. The results showed a lower burst effect and sustained release profile from the hydrogel containing n-propyl gallate-loaded SLN, when compared to the n-propyl gallate-loaded SLN alone. In addition, the cumulative permeation of n-propyl gallate from the hydrogel was 3- to 60-fold higher than that of n-propyl gallate-loaded SLN alone and native n-propyl gallate, respectively.

Sabrina Knoke and Heike Bunjes [9] investigated the release of poorly water-soluble drugs from nanoemulsions for intravenous administration in release media containing components that mimic physiological acceptors in vivo. In this study, the transfer of fenofibrate, retinyl acetate and orlistat from nanoemulsion droplets to lipid-containing hydrogel particles that mimic lipoproteins was investigated. Additionally, the transfer of the same drugs from nanoemulsion droplets to bovine serum albumin was investigated. The results showed a slower transfer rate for the lipid-containing hydrogel particles to the highest logP drugs. Thereby, the researchers suggested using lipid-containing hydrogel particles as a useful tool to compare different lipophilic acceptors to assess drug release from colloidal systems. In contrast, albumin was not relevant as a lipophilic acceptor for the drugs studied.

Xin Guo et al. [10] tested the efficacy of novel lipids to improve the activity of doxorubicin-loaded liposomes against solid tumours. In this study, three lipids containing imidazole groups were incorporated into the membrane of liposomes coated with polyethylene glycol (PEG), creating pH-sensitive convertible liposomes. The results demonstrated that imidazole lipids trigger a greater release of doxorubicin from liposomes conjugated with phosphatidylethanolamine and PEG. Thus, the researchers suggested that the use of pH-sensitive convertible liposomes that balance tissue penetration, cell binding and drug release, would induce ideal activity against solid tumours.

Aziz Unnisa et al. [11] used a three-factor, three-level Box–Behnken design to optimize dapagliflozin-loaded SLN for oral administration. The effectiveness of the optimized formulation for the management of type 2 diabetes, by reducing blood glucose levels, was tested in streptozotocin-induced diabetic rats. The results showed a two-fold increase in oral drug absorption, when compared to a commercial formulation of pure dapagliflozin.

Maria Carmo Pereira et al. [12] evaluated in vivo the toxicity of multi-dose intravenous administration of neutral liposomes and cationic liposomes for drug delivery. The results showed that the administration of 10 doses of cationic liposomes resulted in a mortality of 45%, while the administration of the same doses of neutral liposomes showed no mortality. From this study, the researchers concluded that neutral liposomes are safe carriers for the administration of repeated doses of drugs.

Rompicherla Narayana Charyulu et al. [13] used a full factorial design to optimize an oral formulation of silymarin-loaded phytosomes, aiming to improve the hepatoprotective activity of the encapsulated compound. The researchers performed in vivo studies in a tetrachloromethane-induced hepatotoxicity rat model and observed a six-fold increase in systemic bioavailability after the oral administration of the optimized silymarin phytosomal formulation, compared to pure silymarin. From these findings, the researchers concluded that phytosomes may be suitable nanocarriers to improve the oral bioavailability of phyto-constituents with poor aqueous solubility.

Shaymaa Wagdy El-Far et al. [14] used a response surface D-optimal factorial design to optimize drug-free niosome formulations, in which model drugs used for colorectal cancer treatment were encapsulated. The amphiphilic characteristics of the niosomes allowed the encapsulation of oxaliplatin (hydrophilic model drug) and placlitaxel (hydrophobic model drug). The results showed that both drugs increased the anticancer activity against HT-29 colon cancer cells, up to two- to threefold, after encapsulation in niosomes, when compared to free drugs. Thus, the researchers concluded that niosomes could be used to improve the therapeutic outcomes of oxaliplatin and paclitaxel against colorectal cancer.

Randa Mohammed Zaki et al. [15] developed a new generation of liposomes containing a high concentration of glycerol, which were called glycerosomes. In this study, the central composite rotatable design was used to optimize an oral formulation of quetiapine fumarate-loaded glycerosomes that showed highly improved brain and plasma drug bioavailability, when compared to an oral drug suspension. From these findings, the researchers proposed the use of quetiapine fumarate-loaded glycerosomes as promising alternative carriers to improve the oral delivery of quetiapine fumarate.

We would like to thank all the authors of this Special Issue for contributing with high-quality works. We also acknowledge all the reviewers, who critically evaluated the articles. In addition, we would like to thank to the Assistant Editor, Ms. Evelyn Du, for her kind help.
